# Association of *PTPN22 *1858 single-nucleotide polymorphism with rheumatoid arthritis in a German cohort: higher frequency of the risk allele in male compared to female patients

**DOI:** 10.1186/ar1945

**Published:** 2006-04-24

**Authors:** Matthias Pierer, Sylke Kaltenhäuser, Sybille Arnold, Matthias Wahle, Christoph Baerwald, Holm Häntzschel, Ulf Wagner

**Affiliations:** 1 Medical Department IV, University of Leipzig, Leipzig, Germany

## Abstract

The functional single-nucleotide polymorphism (SNP) of the gene *PTPN22 *is a susceptibility locus for rheumatoid arthritis (RA). The study presented here describes the association of the *PTPN22 *1858T allele with RA in a German patient cohort; 390 patients with RA and 349 controls were enrolled in the study. For 123 patients, clinical and radiographic documentation over 6 years was available from the onset of disease. Genotyping of the *PTPN22 *1858 SNP was performed using an restriction fragment length polymorphism PCR-based genotyping assay. The odds ratio to develop RA was 2.57 for carriers of the *PTPN22 *1858T allele (95% confidence interval (CI) 1.85–3.58, *p *< 0.001), and 5.58 for homozygotes (95% CI 1.85–16.79). The *PTPN22 *1858T allele was significantly associated not only with rheumatoid factor (RF) and anti-cyclic citrullinated peptide (CCP) positive RA, but also with RF and anti-CCP negative disease. The frequency of the *PTPN22 *1858T allele was increased disproportionately in male patients (53.8% compared to 33.0% in female patients, *p *< 0.001), and the resulting odds ratio for male carriers was increased to 4.47 (95% CI 2.5–8.0, *p *< 0.001). Moreover, within the male patient population, the rare allele was significantly associated with the *HLA-DRB1 *shared epitope (*p *= 0.01). No significant differences in disease activity or Larsen scores were detected. The results provide further evidence that the *PTPN22 *1858T allele is associated with RA irrespective of autoantibody production. The increased frequency of the risk allele in male patients and its association with the shared epitope indicate that the genetic contribution to disease pathogenesis might be more prominent in men.

## Introduction

Rheumatoid arthritis (RA) is a complex autoimmune disease with a strong genetic contribution to its pathogenesis. Studies on twins have shown concordance rates between 12% and 15% in monozygotic twins compared to 4% in dizygotic twins [1]. Calculations based on these data have estimated an overall heritability of about 60% [2,3], indicating that genetic factors account for the majority of population susceptibility to RA.

The *HLA-DRBI *locus accounts for approximately one-third of the genetically determined susceptibility to the disease [4]. The identification of further RA susceptibility loci, both in candidate gene approaches and genome-wide linkage studies, was hindered in the past by difficulties to replicate such results in other study populations. Recently, however, an association between the minor allele (T) of a missense single-nucleotide polymorphism (SNP; R620W (rs2476601, 1858C/T)) in the protein tyrosine phosphatase non-receptor type 22 gene (*PTPN22*) and susceptibility to RA has been described [5]; this has been confirmed in several large cohorts of patients and controls [6-18]. Besides its association with RA, the *PTPN22 *1858T allele has been found to be associated also with type I diabetes, systemic lupus erythematosus and autoimmune thyroiditis, suggesting a genetic predisposition towards generalized T cell autoimmunity. The missense SNP lies within the first proline-rich domain of PTPN22 and results in the substitution of tryptophan for arginine at codon 620 (R620W) of PTPN22. The *PTPN22 *1858T variant has recently been described to result in a gain-of-function form of the enzyme [23], leading to stronger suppression of the early T cell activation process. Possible pathogenetic mechanisms implied by this finding include failure to delete autoreactive T cells during thymic selection or decreased activity of regulatory T cells.

The aim of this study was to analyze the association of the 1858C/T SNP with RA in a sample set comprising 390 German white RA cases and 349 healthy German white controls. In addition, the influence of the *PTPN22 *1858T allele on the clinical course of the disease and its relationship to gender, *HLA DRB1*, the presence of rheumatoid factor (RF) and anti-cyclic citrullinated peptide (CCP) antibodies was explored.

## Materials and methods

### RA-susceptibility cohort and control subjects

DNA was available from 390 RA patients and 349 population control subjects. All RA patients satisfied the 1987 American College of Rheumatology criteria for RA [24]. Of the RA patients, 83.3% had demonstrated erosions and 74.6% were RF positive. The median age at onset of RA was 47.0 years (interquartile range 37 to 59 years), the median disease duration was 12.5 years (interquartile range 9 to 22 years) and 76.1% of the RA patients were female. *HLA DRB1 *high-resolution genotyping was performed to define the shared epitope (SE) positive DRB1*04 alleles *0401, *0404, *0405 and *0408. In the ethnically very homogenous German population, the alleles DRB1*0102 and *0103 are extremely rare. Therefore, all individuals positive for DRB1*01 in low resolution typing were considered SE positive, as were all DRB1*10 positive individuals. DRB1*1402 did not occur in the study population. HLA genotyping revealed that 30.3% of the patients had zero copies, 42.6% had 1 copy, and 27.1% had 2 copies of the shared epitope.

Control subjects with no history of inflammatory arthritis were recruited among healthy blood donors and from general practice registers. All patients and controls were white subjects of German origin without discernable ethnic variation. Control individuals were recruited with ethics committee approval and provided their informed consent. Among the 349 population-based control subjects, 223 (63.6%) were female and 57.3% had zero copies, 35.3% had 1 copy, and 7.4% had 2 copies of the SE.

The subgroup of patients (*n *= 123) who were analyzed for the influence of the *PTPN22 *1858 SNP on radiographic progression was recruited in the outpatient clinic of the Department of Medicine IV, Leipzig University, as part of a long term prospective observational study. RA patients with recent onset RA were enrolled at the first presentation to a rheumatologist. The median disease duration before study enrollment was six months. This study population was partially overlapping with patient cohorts reported previously, and the clinical and immunogenetic characteristics did not differ from those earlier reports [25,26].

### Genotyping methods

For genotyping, cellular DNA was isolated from 10 ml of peripheral blood using standard procedures, and 0.5 μg DNA were used in the PCR reactions. For the determination of the *PTPN22 *alleles, PCR-based restriction fragment length polymorphism (RFLP) analysis was performed as described previously [20]. Briefly, a fragment of the *PTPN22 *gene was amplified by PCR using the forward primer 5'-TCA CCA GCT TCC TCA ACC ACA-3' and the reverse primer 5'-GAT AAT GTT GCT TCA ACG GAA TTT A-3'. The C→T transition at codon 620 (NCBI refSNP ID: rs2476601) creates in the 1858T allele a restriction site for Xcm I. The polymorphism was identified by Xcm I (New England Biolabs, Beverly, MA, USA) restriction endonuclease digestion of the PCR amplified fragment. Each digestion was resolved on 3% agarose gel, stained with ethidium bromide and visualized by UV. Repeated typing was performed in 10% of patient samples, with identical results in all cases.

The genotype obtained by RFLP-PCR assay was verified in 12 randomly selected samples from each genotype by direct sequencing using the same primers (ABI 7000; Applied Biosystems, Foster City, CA, USA), and was confirmed in all instances.

For *HLA-DRB1 *typing, genomic DNA was PCR amplified using two primers specific for the second exon of *DRB1 *as described previously [26]. Low resolution typing of *DRB1 *specificities was performed by oligonucleotide hybridization of the PCR products to probes specific for DRB1*01 through *18. Hybridization was performed in a dot-blot format with digoxigenin-11-ddUTP-labeled oligonucleotides. After the stringent wash, detection was carried out using anti-digoxigenin antibody-alkaline phosphatase conjugate (Boehringer Mannheim, Mannheim, Germany) and CSPD (disodium 3-(4-methoxyspiro (1,2-dioxetane-3,2-(5'-chloro)tricyclo [3.3.1.13,7]decan)-4-yl)phenyl phosphate; Tropix, Bedford, USA) as chemiluminescent substrate. For DRB1*04 subtyping, primers and oligonucleotides were used as published previously [26].

### Rheumatoid factor determination

RF values were determined by laser nephelometry according to the manufacturer's instructions (Dade Behring, Liederbach, Germany). In more than 90% of patients, repeated RF measurements were available. Individuals with values ≥40 IU/ml on at least one occasion were regarded as RF positive since this cutoff has been established by the central laboratory facility and is recommended for routine clinical use.

### Detection of anti-CCP antibodies

A commercially available, second generation anti-CCP ELISA (Immunoscan RA2, Generic Assays, Dahlewitz, Germany) was used for the quantification of anti-CCP antibodies in patient sera. A cut off of 50 units/ml was used as a stringent criterion for anti-CCP antibody positivity.

### Statistical analysis

Allele and genotype frequencies of *PTPN22 *1858T were obtained by direct counting. For allele and genotype comparisons, the chi-square test was used. Odds ratios (ORs) and 95% confidence intervals (95% CIs) were calculated according to Woolf's method. Differences in medians or means between groups were analyzed using Mann-Whitney or *t* test where appropriate. Multiple logistic regression analysis was performed to determine the influence of different genetic variables. The software used was the Sigmastat program (Systat 2004, Richmond, California, USA).

## Results

Genotype frequencies for the *PTPN22 *1858T SNP were in Hardy-Weinberg equilibrium in both the patient and the control cohort and all analyzed subgroups. The distribution of genotypes and the resulting allele frequencies of the variant *PTPN22 *1858T allele in RA patients and healthy controls are shown in Table 1.

All genotypes containing the rare 1858T allele were found at increased frequencies in RA patients. The CT and TT genotype was present in 37.9% of patients and 19.2% of healthy controls, resulting in an OR of 2.57 (95% CI 1.85–3.58, *p *< 0.001). Carriage of a homozygous TT genotype was associated with an even higher OR, supporting a gene dosage effect for the *PTPN22 *1858 SNP.

For the analysis of differential associations of the *PTPN22 *1858 SNP in clinically and immunogenetically defined subgroups, the RA patients were stratified for several parameters. The ORs indicated in Table 2 show a significant association of the risk allele genotypes with both RF positive and RF negative RA (ORs for CT and TT genotype were 2.67 and 2.3, respectively).

Stratification of the patients for anti-CCP antibody positivity showed a similar association of *PTPN22 *1858T alleles with RA, irrespective of the presence of anti-CCP antibodies (ORs for CT and TT genotype were 2.62 and 2.63, respectively; Table 2).

Since genetic interactions between *HLA *and non-*HLA *loci have been described for susceptibility to RA and other autoimmune diseases [27], genotype distributions for the *PTPN22 *1858T SNP in subgroups stratified according to the number of *HLA-DRB1 *SE alleles were determined and compared to controls. In line with results reported previously, the presence of the *HLA-DRB1 *SE was found to have no effect on the association of the *PTPN22 *1858T allele with the disease, since the frequencies of the *PTPN22 *1858T allele in RA patients and controls and the resulting ORs in the subgroups with zero, one or two copies of the shared epitope were comparable (OR 2.15, *p *= 0.007; OR 2.59, *p *< 0.001; and OR 2.0, *p *= not significant). The loss of significance in the subgroup analysis of SE homozygous individuals is explainable by the small sample size.

Stratification of patients and controls for gender showed a significant association of the *PTPN22 *1858T allele with RA in both male and female patients compared to the appropriate controls (Table 3). However, the frequency of a *PTPN22 *1858T genotype was significantly higher in male patients compared to female patients (53.8% versus 33%, *p *< 0.001; resulting ORs 4.47 and 2.19, respectively). In male patients, an additional influence of the RA associated *DRB1 *SE was discernible. The frequency of the 1858T allele was significantly higher in the SE positive subgroup compared to the SE negative patients (62.3% versus 29.2%; *p *= 0.01, power of the χ^2 ^test with α = 0.73 below the desired level).

To analyze the independent contribution of the genetic covariates to disease risk, multivariate analysis was performed. When the presence of SE, homozygosity for SE, the presence of the 1858T allele and homozygozity for the 1858T allele were entered in a multiple logistic regression analysis, all of the covariates with the exception of *PTPN *1858T homozygosity exerted significant influences on the disease risk (OR 2.19, *p *< 0.001; OR 2.80, *p *< 0.001; OR 2.13, *p *< 0.001; OR 2.91, *p *= 0.10). In a second, separate analysis, the presence of RA associated DRB1*04 alleles and DRB1*01 alleles was entered in addition to the 1858T allele, while SE status was not included. In this logistic regression, both RA associated DRB1 specificities exerted independent significant influences on the disease risk (OR 3.23, *p *< 0.001 for DRB1*04; OR 1.95, *p *< 0.001 for DRB1*01), while the 1858T allele retained its significant impact (OR 2.22, *p *< 0.001).

Comparison of clinical and demographic features in *PTPN22 *1858T allele positive and negative RA cases showed no differences in the frequency of SE carriership, the concentrations of anti-CCP antibodies, the concentrations of IgM RF, the concentrations of IgA RF or the presence of erosions (data not shown). However, the median age at disease onset of patients carrying a *PTPN22 *1858T allele was 5.5 years younger compared to patients without such a genotype (CT and TT genotype 45.5 years versus CC genotype 51.0 years; *p *= 0.029), in line with a previously published report [9]. No difference in the disease duration between both groups was discernable (*p *= 0.69).

For a subgroup of the patient population, complete clinical documentation was available starting from the first presentation in a rheumatology department, because they had been part of a previously described prospective study for six or more years [25,26]. In those 123 patients, the influence of the *PTPN22 *1858T genotype on the clinical course of the disease, including the radiographic progression, was investigated. C-reactive protein and erythrocyte sedimentation rate did not differ between the groups defined by the *PTPN22 *1858 SNP. In addition, no differences in the number of swollen joints at study entry and after two years were observed (data not shown).

The analysis of the progression of erosive joint destruction was performed by comparing Larsen scores determined prospectively at the indicated time points. No significant differences in the Larsen scores were found between the groups positive and negative for the *PTPN22 *1858T allele. There was a non-significant trend, however, in the positive patients towards higher Larsen scores at several of the analyzed time points, but not at study entry (Table 4).

## Discussion

The study presented confirms the association of the *PTPN22 *1858T allele with RA in a study cohort of white Germans. The genotype and allele frequencies in the healthy controls were comparable to those reported previously in populations from the US, Canada and Spain, and nearly identical to data reported from Great Britain and New Zealand [5-10]. However, the carrier frequency of the 1858T allele in RA patients was higher than in some of the published cohorts [5,7,8,10], resulting in a somewhat higher OR for carriers to develop RA. Possible explanations include random fluctuation and differences in sample bias because the analyzed patient cohort is ethnically homogenous, was not recruited in a multi-center study, and is characterized by long disease duration before analysis.

In the first study showing association of the variant *PTPN22 *SNP with autoimmune disease, a gene dosage effect has been suggested, since individuals homozygous for the *PTPN22 *1858T allele were most likely to develop type I diabetes [20]. Our data, together with other reports, support this hypothesis since a substantially higher susceptibility to RA was found for individuals who were homozygous for the 1858T allele.

One important finding of the initial report about *PTPN22 *1858 SNP as a RA susceptibility locus was the limitation of this association to RF positive RA [5]. Subsequently, the *PTPN22 *1858T allele was found to be associated with RF negative disease in several studies [7,8,10,13], while other studies reproduced the lack of association with RF negative disease [9,11,15,18]. In the study cohort analyzed here, the *PTPN22 *1858T allele was associated not only with RF negative disease, but also with anti-CCP negative RA, which is in contrast to two previous studies [11,18]. Sample bias as well as ethnic differences might contribute to these discrepancies. It needs to be emphasized, however, that our data confirm the association of the *PTPN22 *1858T allele with RF and anti-CCP negative RA in a patient cohort with longstanding disease and repeated RF and anti-CCP measurements, which makes it unlikely that conversion to RF positive and anti-CCP positive status could occur in carriers of the risk allele at later stages of the disease.

An interesting finding of the study presented here is the disproportionately high frequency of the *PTPN22 *1858T allele in male patients suffering from RA, which results in a higher risk to develop RA for male compared to female carriers. In the case control study from Spain, the risk allele was also observed in a higher frequency in male cases, but significance was lost after correction for multiple statistical testing [7]. However, a recent study of more than 4,000 patients from North America and Sweden has also demonstrated a significantly stronger effect of *PTPN22 *in males than females [18]. Different immunogenetic associations in male and female RA patients have also been described for several *HLA DRB1 *alleles [28,29]. One possible explanation of these findings is that male and female RA are partially diverging disease entities, which has been suggested previously based on clinical observations [30]. Alternatively, environmental influences like smoking, which are regionally more frequently present in men [31], might contribute to a higher rate of disease development on a genetic background with a given disease susceptibility. Of possible relevance in this context is the association between the presence of the *HLA DRB1 *SE and the presence of the *PTPN22 *1858T allele exclusively in male patients in our study. However, the number of patients in this subgroup was small and the phenomenon has not been observed in previous study cohorts with larger numbers of male patients and, therefore, warrants further investigation.

With regards to the clinical implications of the presence of the *PTPN22 *1858T allele as a prognostic marker applicable in the clinical management in early stages of the disease, no significant differences were discernible. Nevertheless, non-significant differences in the course of Larsen scores over six years of observation indicate that a detailed analysis in larger prospectively followed patient cohorts might yield significant results. In a patient cohort from the UK, influences on disease severity have indeed been reported [9], although no such influence was observed in a recent study of an inception cohort with documented radiographic progression over four years [11].

## Conclusion

The association of RA with a missense SNP in the gene *PTPN22 *could be replicated in a German population. The *PTPN22 *1858T allele was found to be associated with RF positive disease, but also with RF negative and anti-CCP antibody negative RA. In the male cohort, the *PTPN22 *1858T allele was present in more than half of the patients, suggesting that RA in men might be a clinically more homogeneous, genetically predetermined condition.

## Abbreviations

CCP = cyclic citrullinated peptides; CI = confidence interval; OR = odds ratio; PTPN22 = protein tyrosine phosphatase non-receptor type 22; RA = rheumatoid arthritis; RF = rheumatoid factor; RFLP = restriction fragment length polymorphism; SE = shared epitope; SNP = single-nucleotide polymorphism.

## Competing interests

The authors declare that they have no competing interests.

## Authors' contributions

MP designed the study, oversaw all aspects of the laboratory work, analyzed the data and prepared the manuscript. SK, SA, MW and CB participated in the collection of clinical data and the recruitment of patients into the study. UW and HH participated in the design of the study, statistical analysis, interpretation of the results, and writing of the final manuscript.

## Figures and Tables

**Table 1:** Genotype and allele frequencies of PTPN22 1858 T/C SNP in controls and patients with RA

	Genotype frequency			Allele frequency
	CT	CT and TT	TT	T

Controls (*n *= 349)	18.3 (64)	19.2 (67)	0.9 (3)	10.0 (70)
RA (*n *= 390)	33.3 (130)	37.9 (148)	4.6 (18)	21.3 (166)
OR (95% CI)	2.23^a ^(1.59–3.13)	2.57^a ^(1.85–3.58)	5.58^b ^(1.85–16.79)	2.43^a ^(1.81–3.26)

Values are the frequency in percent with absolute number of genotypes or alleles given in parentheses. Odds ratios (OR), 95% confidence intervals (CI) and p values are for the comparison of frequencies of the indicated markers in the patients compared to the controls. Level of significance: ^a^*p *< 0.001 and ^b^*p *< 0.005, power for all calculations α > 0.8.

**Table 2:** Genotype frequencies of *PTPN22 *1858 T/C SNP in RA patients, stratified by rheumatoid factor and anti-CCP antibody status

	CT	CT and TT	OR for CT and TT (95% CI)
RF+ (*n *= 291)	34.7 (101)	38.8 (113)	2.67^a ^(1.88–3.79)
RF- (*n *= 99)	29.3 (29)	35.4 (35)	2.3^a ^(1.42–3.72)
CCP+ (*n *= 193)	34.7 (67)	38.3 (74)	2.62^a ^(1.78–3.86)
CCP- (*n *= 65)	35.4 (23)	38.5 (25)	2.63^a ^(1.52–4.56)

Values are the frequency in percent with absolute number of genotypes given in parentheses. Rheumatoid arthritis (RA) patients (*n *= 390) were stratified by rheumatoid factor (RF) status (values ≥40 IU/ml were regarded as RF positive (RF+)). RA patients for whom anti-cyclic citrullinated peptide (CCP) antibody concentrations were available (*n *= 258) were stratified according to anti-CCP positivity (values ≥50 IU/ml were regarded as CCP positive (CCP+)). The odds ratios (ORs) and 95% confidence intervals (95% CIs) were calculated in the indicated patient subgroups in comparison to controls. Level of significance: ^a^*p *= 0.001, power for all calculations α > 0.9.

**Table 3:** Comparison of genotype frequencies for *PTPN22 *1858 SNP in patients with RA and controls stratified by sex

	CT	CT and TT	OR for CT and TT (95% CI)
Female			
Controls (*n *= 223)	17.9 (39)	18.4 (41)	-
RA (*n *= 297)	28.6 (85)	33.0 (98)	2.19^a ^(1.45–3.31)
Male			
Controls (*n *= 126)	19.8 (25)	20.6 (26)	-
RA (*n *= 93)	48.4 (45)	53.8 (50)	4.47^a ^(2.51–7.96)

Values are the frequency in percent with absolute number of genotypes given in parentheses. Rheumatoid arthritis (RA) patients and controls were stratified by sex, and odds ratios (ORs) and 95% confidence intervals (95% CIs) were calculated for carriage of the PTPN22*T allele in comparison to controls. Level of significance: ^a^*p *< 0.001, power for all calculations α > 0.95.

**Table 4:** Radiographic progression in RA patients according to the presence or absence of the *PTPN22 *1858T allele

	CC (*n *= 78)	CT/TT (*n *= 45)	*P* value
Larsen score at study entry	0 (0–2.5)	0 (0–4.7)	0.612
Larsen score after 1 year	2.0 (0–12.2)	11.5 (0–20.0)	0.061
Larsen score after 2 years	10.0 (0–26.0)	18.5 (2.0–34.5)	0.076
Larsen score after 4 years	16.0 (2.0–30.0)	24.0 (4.7–41.5)	0.136
Larsen score after 6 years	22.0 (10.7–39.2)	37.0 (15.25–52.7)	0.159

Data are medians and interquartile range (IQR) for the comparison between *PTPN22 *risk allele negative and positive prospectively followed patients (*n *= 123). Levels of significance are given as determined by univariate analysis, power for all comparisons α < 0.8.
